# Neurocysticercosis, a Persisting Health Problem in Mexico

**DOI:** 10.1371/journal.pntd.0000805

**Published:** 2010-08-24

**Authors:** Agnès Fleury, Jael Moreno García, Paulina Valdez Aguerrebere, María de Sayve Durán, Paola Becerril Rodríguez, Carlos Larralde, Edda Sciutto

**Affiliations:** 1 Instituto Nacional de Neurología y Neurocirugía, Mexico City, México; 2 Instituto de Investigaciones Biomédicas, Universidad Nacional Autónoma de México, Mexico City, México; Center for Disease Control, United States of America

## Abstract

**Background:**

The ongoing epidemiological transition in Mexico minimizes the relative impact of neurocysticercosis (NC) on public health. However, hard data on the disease frequency are not available.

**Methodology:**

All clinical records from patients admitted in the Instituto Nacional de Neurologia y Neurocirugia (INNN) at Mexico City in 1994 and 2004 were revised. The frequencies of hospitalized NC patients in neurology, neurosurgery and psychiatry services, as well as NC mortality from 1995 through 2009, were retrieved. Statistical analyses were made to evaluate possible significant differences in frequencies of NC patients' admission between 1994 and 2004, and in yearly frequencies of NC patients' hospitalization and death between 1995 and 2009.

**Principal Findings:**

NC frequency in INNN is not significantly different in 1994 and 2004. Between these two years, clinical severity of the cases diminished and the proportion of patients living in Mexico City increased. Yearly frequencies of hospitalization in neurology and psychiatry services were stable, while frequencies of hospitalization in neurosurgery service and mortality significantly decreased between 1995 and 2009.

**Conclusions:**

Our findings show a stable tendency of hospital cases during the last decade that should encourage to redouble efforts to control this ancient disease.

## Author Summary

Human neurocysticercosis is a severe parasitic disease caused by the installation of *Taenia solium* larvae in the central nervous system. Neurocysticercosis is still deeply rooted in Latin-America, Africa and Asia, where it develops its complete life cycle promoted by poor sanitary conditions. It is also emerging in developed countries due to human migration. Although hard data on the evolution of the disease incidence in endemic countries are lacking, its presence is being obscured by the growth of degenerative and metabolic diseases, creating the illusion of having disappeared.

In this article, we show that neurocysticercosis frequency has not significantly changed between 1994 and 2009 among patients attending the Instituto Nacional de Neurología y Neurocirugía, Mexico City, the principal Mexican neurological center. We also show that clinical severity of the cases diminished during this period, associated with the higher proportion of neurocysticercotic patients from Mexico City rather than from the states, where local neurological facilities have improved. These results show that neurocysticercosis is still relevant in México, and that more effective efforts should be put toward its eradication.

## Introduction

Neurocysticercosis (NC) is a life-threatening and costly parasitic disease, endemic in most non-developed countries and increasing in developed world [1]–[5]. NC real prevalence and incidence are difficult to assess, as symptoms are highly heterogeneous and its diagnosis requires neuroradiological studies, not available to all population at risk. Anyway, some data show the persistence of active transmission in Mexico. Particularly, a partial report including only patients hospitalized at INNN, Mexico, showed no statistically significant decrease of NC frequency between 1995 and 2001 (from 2.4 to 1.8%) [6], transversal surveys in rural communities indicate the persistence of human NC (prevalence>9%) [7], [8] and porcine cysticercosis in rural pigs (up to 30%) [9], [10]. In spite of these data, the epidemiological transition occurring in Mexico, with increased diagnosis of metabolic, neoplastic and degenerative diseases [11], could lead us to disregard the importance of NC in Mexico [12]. Herein, the frequency of NC in all patients who attended at INNN in 1994 was compared with that of 2004, and frequencies of hospitalization and mortality of NC patients in neurology, neurosurgery and psychiatry services between 1995 and 2009 are presented.

## Materials and Methods

This study was performed at the Instituto Nacional de Neurología y Neurocirugía (INNN). INNN is a public, third-level referral center located in Mexico City, where neurological, neurosurgical and psychiatric patients above 15 years old are attended. Admission is reserved to patients lacking social security.

To evaluate the evolution of NC burden on INNN, two approaches were taken. First, all clinical records of patients admitted at INNN in 1994 (*n* = 4098) and 2004 (*n* = 4706) were manually, anonymously reviewed. Patients fulfilling criteria of NC definitive diagnosis based on Del Brutto's et al. criteria [13] were selected and their demographical, clinical and radiological characteristics were obtained. Second, the yearly numbers of hospitalized NC patients and deaths due to NC at neurology, neurosurgery and psychiatry services from 1995 to 2009 were obtained from INNN's epidemiological service. Frequencies of NC hospitalized patients and NC mortality with respect to the total number of hospitalized patients in each service were calculated.

Statistical analysis using SPSS software was completed. Chi-square test was made to evaluate statistical differences between proportions and Student t-test between means. 95% confidence intervals of proportions and means were provided. Linear regression was used to test changes during calendar periods, considering calendar year as the independent variable.

This study was approved by INNN Institutional Review Board.

## Results

Table 1 summarizes the differences in the frequency of NC cases attended in the INNN in 1994 and 2004. Between the years 1994 and 2004, no significant statistical differences were found neither in the frequency of NC at INNN (100/4098, 2.4% vs. 120/4706, 2.5%, respectively) nor in patients general profile. Two significant differences were observed, though: in 2004, the cases severity (presence of intracranial hypertension) significantly decreased (*P* = 0.007), and the proportion of patients living in Mexico City significantly increased (*P* = 0.005).

**Table 1:** Clinical features of NC patients in 1994 and 2004.

	1994	2004	P
NC frequency	2.4 (2.0–2.9)	2.5 (2.1–3.0)	0.74
Age (years)^‡^	38.1 (35.5–40.8)	38.8 (36.2–41.5)	0.72
Sex (% Male)	53 (42.7–63.1)	51.7 (42.4–60.9)	0.84
City of origin (% Mexico City)	20 (0.13–0.29)	37.5 (0.29–0.47)	0.005
Symptoms at diagnosis			
Headache	12 (6.3–20)	18.3 (11.8–26.4)	0.2
Seizures	36 (26.6–46.2)	35.8 (27.2–45.0)	0.98
Intracranial hypertension	45 (35.1–55.3)	27.5 (19.7–36.4)	0.007
Radiological picture*			
Parasite viability (% viable)	56.7 (46.2–66.7)	59.6 (50.1–68.7)	0.66
number (% multiple)	58.8 (48.3–68.7)	63.2 (53.6–72.0)	0.51
localization (% SAb or V)	55.7 (45.2–65.8)	45.6 (36.3–55.2)	0.14
Cellularity of CSF (cells/mm^3^)^ ‡,^ ***	24.5 (12.1–36.9)	26.5 (16.4–36.5)	0.80

All values are proportions and expressed as percentages, unless designated as a mean(^‡^). 95% confidence intervals are presented in parentheses.

*: 9 patients did not harbor a cysticercus at the time of diagnosis in INNN, some of them only showed arachnoiditis and others had been treated before in other hospitals.

**: SAb: SubArachnoidal space of the base of the skull/V: Ventricular/P: Parenchymal/Sas: SubArachnoidal space of the sulci.

***: Lumbar puncture was performed at time of diagnosis in only 79 NCC patients in 1994, and in 79 patients in 2004.

**Figure 1:**
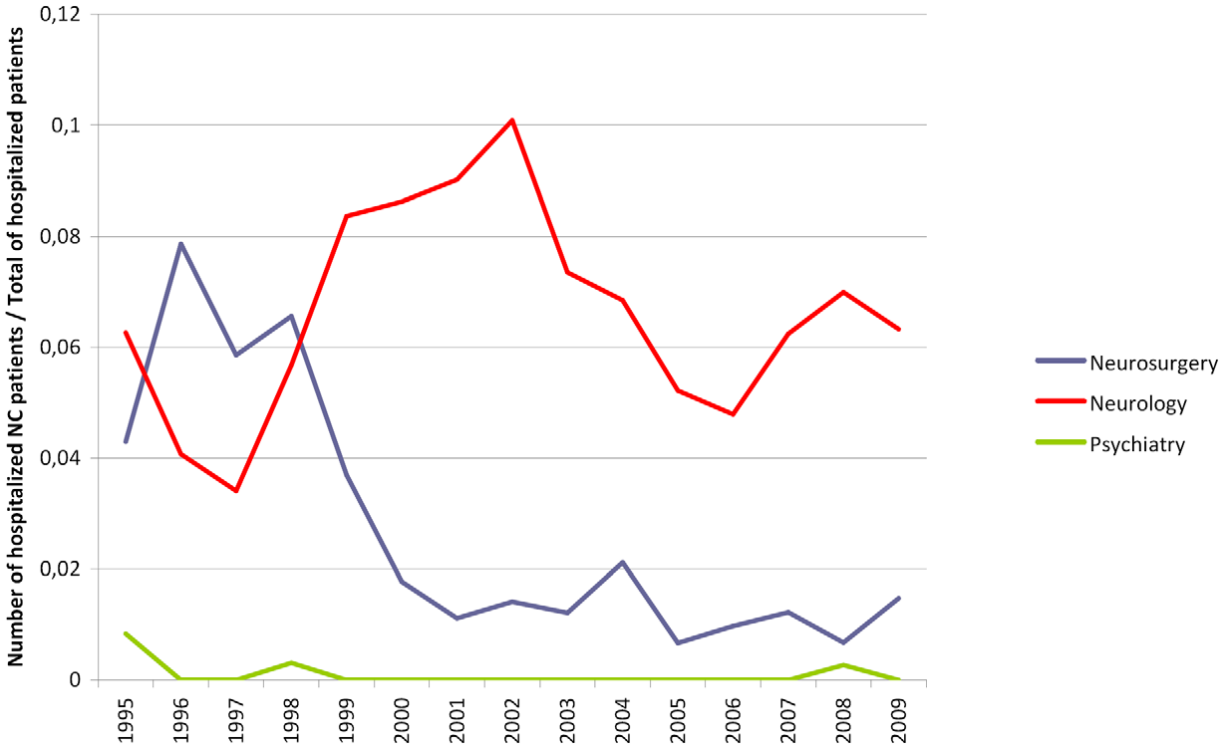
Annual frequencies of hospitalized NC patients (INNN, 1995–2009).

Figure 1 shows the evolution of NC frequency in hospitalized patients between 1995 and 2009. In neurosurgery service, frequencies of hospitalization of NC patients varied between 0.67 and 7.9% with a statistically significant decrease between these two dates (r = –0.79, P<0.001). In neurology service, frequencies of hospitalization varied between 3.4 and 10.9% without any significant temporal trend (r = 0.12, P = 0.67), while in psychiatry, only five NC patients were hospitalized during the 15 years under study, with no significant tendency (*r* = –0.38, *P* = 0.16). Concerning mortality during this period, 28 NC patients died at INNN, with a significant decrease in mortality frequency (*r* = –0.7, *P* = 0.003).

## Discussion

The evident epidemiological transition occurring in Mexico, with growing incidence of metabolic, neoplastic, and degenerative diseases [11], sometimes leads us to forget the weight of other “archaic”, infectious disorders, linked mainly with poverty. NC is one of those. Evolution of NC prevalence is not known, as population-based studies are not available. In the present report, during the 10-year period studied the frequency of NC showed no significant decrease in INNN, the most active neurological center in Mexico. However, some significant changes did occur. The proportion of NC patients from Mexico City increased, possibly due to rural population's migration. The acquisition of computerized tomography, between 1994 and 2004, by most of the public hospitals in States neighboring Mexico City may also have resulted in more frequent local NC diagnosis than before and in lessening their attendance to INNN. The only optimistic change detected in this study was the significant decrease in the severity of NC, a change that could be related with its earlier diagnosis. These results were confirmed when only hospitalized patients were considered between 1995 and 2009: the frequency of hospitalized patients in neurology was stable, while NC frequency in neurosurgery service, as well as NC mortality, decreased.

These data strengthen the notion of persistent active transmission, in agreement with the high prevalence of pig cysticercosis (13.3%) reported in recent epidemiological studies performed in rural areas of Mexico [14]. The latter is the most appropriate indicator to demonstrate active transmission, since 80% of pigs in rural communities are consumed before they are one-year-old [10]. In rural areas, the high prevalence of pig cysticercosis is accompanied by a high frequency of human calcified neurocysticercosis cases and an extremely low frequency of vesicular cysticerci [8], [15]. These disproportions between calcified and vesicular cysticerci in human NC indicate that in most cases the parasite is destroyed. On the other hand, the stability of NC frequency in INNN also indicate that, despite this fact, the parasite (vesicular or calcified) still causes symptoms in an important number of infected subjects, resulting in around 2.4% of consultations in INNN are due to NC.

This study demonstrates that NC is still a health problem of Mexico. It is important to note that INNN and Mexico City does not properly represent the whole country. However, the stability of NC frequency in patients attending INNN and in the hospitalization rate in the INNN neurology service must alert medical practitioners and health authorities on the persistence of unresolved health problems related with poverty. Efforts should be encouraged to apply effective measures for their eradication.
